# Characterization of Diffusion Metric Map Similarity in Data From a Clinical Data Repository Using Histogram Distances

**DOI:** 10.3389/fnins.2018.00133

**Published:** 2018-03-08

**Authors:** Graham C. Warner, Karl G. Helmer

**Affiliations:** ^1^Athinoula A. Martinos Center for Biomedical Imaging, Charlestown, MA, United States; ^2^Department of Radiology, Massachusetts General Hospital, Boston, MA, United States; ^3^Harvard Medical School, Harvard University, Boston, MA, United States

**Keywords:** histogram distance, diffusion MRI, diffusion tensor imaging, data quality, data reproducibility

## Abstract

As the sharing of data is mandated by funding agencies and journals, reuse of data has become more prevalent. It becomes imperative, therefore, to develop methods to characterize the similarity of data. While users can group data based on the acquisition parameters stored in the file headers, these gives no indication whether a file can be combined with other data without increasing the variance in the data set. Methods have been implemented that characterize the signal-to-noise ratio or identify signal drop-outs in the raw image files, but potential users of data often have access to calculated metric maps and these are more difficult to characterize and compare. Here we describe a histogram-distance-based method applied to diffusion metric maps of fractional anisotropy and mean diffusivity that were generated using data extracted from a repository of clinically-acquired MRI data. We describe the generation of the data set, the pitfalls specific to diffusion MRI data, and the results of the histogram distance analysis. We find that, in general, data from GE scanners are less similar than are data from Siemens scanners. We also find that the distribution of distance metric values is not Gaussian at any selection of the acquisition parameters considered here (field strength, number of gradient directions, *b*-value, and vendor).

## Introduction

Data sharing has been promoted as a way to accelerate advances in neuroscience and test the reproducibility of reported results (Poline et al., 2012). As data-sharing requirements become prevalent and more data becomes available, it is of interest to ask: how similar is the data in a repository and can the data be meaningfully combined? We describe here a method based on histogram distances that we apply to diffusion MRI data retrieved from a clinical data repository. Previously (Helmer et al., 2016), we have applied this method to diffusion-metric maps that were collected during a multi-site study of diffusion reproducibility; data that was acquired using an imaging protocol that was harmonized across the three major MRI vendors (Siemens, Philips, and GE) and using a protocol suggested by Landman et al. (2007) and Farrell et al. (2007). In that study, the goal was to identify the effect size of differences in field strength, vendor, site, and echo time on diffusion metric maps (fractional anisotropy, FA, and mean diffusivity, MD). This method determined which parameters most strongly affected the similarity of the resulting maps in a uniformly-acquired data set. That work employed an experimentally-determined threshold for statistical significance for the histogram distances through simulations of histograms of a known distribution. There we found that the thresholds determined *in silico* were reasonable and able to separate smaller effects like site, from larger effects like echo time. In the present study, we examine the ability of histogram distance metrics to characterize the similarity of a heterogeneous clinical data set of the type available from publicly-available data repositories. Repository data is an important use case because it can be used as either a supplement to locally-acquired data in order to increase statistical power or as a normative data set against which to compare data acquired in other studies of unknown quality.

Diffusion-weighted MRI data in a clinical repository may originate from multiple vendors, sites, scanners with different field strengths and be collected using protocols that differ in *b*-value, number of gradient directions, number of T2-weighted scans (*b* = 0 scans) as well as acquisition parameters such as bandwidth, voxel-size, and echo time. Choices of values for each of these can affect the calculated metric maps (e.g., MD and FA). These parameters can also affect the reproducibility of data sites in a multi-site study (Cercignani et al., 2003; Pfefferbaum et al., 2003; Pagani et al., 2010; Fox et al., 2012; Magnotta et al., 2012; Takao et al., 2012), but there, effort is expended to harmonize the acquisition protocols as much as possible to minimize differences between data sets in signal- and contrast-to-noise ratios, image distortion, and pools of spins that contribute to the observed signal. In the case of a clinical repository, the protocol will vary by intent, usually depending upon the presented symptoms of the patient and so, some method is needed to characterize the similarity of data before it can meaningfully be reused. Note that this method is not specific to diffusion-weighted maps, it can be applied to any type of images or maps, though with images, the image intensity values can differ greatly due to differences in gain and scaling.

### Histogram distance

To compare results derived from data collected on different hardware with different protocols, metrics are needed to characterize the degree of similarity. One approach to the comparison problem is to use calculated metrics based on the distance between histograms of image intensity values. This is regularly done in fields as diverse as flow cytometry (Cox et al., 1988; Bernas et al., 2008) and image feature matching (Duda et al., 2000; Haibin and Kazunori, 2006). Histogram distance measures are also often used to quickly search for images within a database (Long et al., 2003) when the incoming data rate is too large to attach metadata to each stored image.

Given the complexity of modeling the underlying mechanisms that result in a given FA or MD map, a general histogram comparison method would be non-parametric and have low computational burden. In addition, there would be some way of assessing the statistical significance of the calculated difference. One commonly used method to find the difference between histograms is the two-sample Kolmogorov–Smirnov (K–S) test (Pettitt and Stephens, 1977; Young, 1977), which estimates the likelihood that two histograms originate from the same, though not necessarily known, continuous distribution by calculating the maximum absolute deviation of the cumulative histograms. The K–S method has been shown to be conservative for binned data (Noether, 1963) and therefore other methods have been proposed. Cox et al., have posited that if the number of counts in a given channel is relatively large (~20), the distribution of the counts is approximately governed by Poisson statistics and hence bin-by-bin confidence intervals and tests can be developed (Cox et al., 1988). Roederer et al. (2001) have noted, however, that Cox et al.'s statistic is weighted toward bins with greater numbers of counts and therefore may not be sensitive to outlier populations. Therefore, they have proposed “probability binning,” which chooses bin widths based on a control sample such that each bin contains an equal number of counts and thus equal statistical weight. Both the Roederer and Cox methods use a modified chi-squared statistic. Probability binning, however, has the drawbacks that it does not satisfy the triangle inequality (see the Appendix) and the binning is dependent upon a particular control sample. It should also be noted that Lampariello (2000) has used the K–S test as a metric for estimating the variability in control samples, which can then be used to give information on whether or not test histograms are significantly different from the control histograms. In *in vivo* studies, however, these methods are not applicable due to the lack of standard samples.

In the field of pattern recognition, many other metrics have been proposed to measure the distance between histograms (Cha and Srihari, 2002). We investigate the efficacy of metrics from seven different families of distance measures. Two broad categories of histogram distance metrics comprise the families of metrics investigated here: those that treat histograms as vectors and those that treat them as probability distribution functions (PDF). The families were (1) Minkowski, (2) Fidelity, (3) Intersection, (4) Inner Product, (5) Squared L2 Norm, (6) Shannon Entropy, and (7) Earth Movers. Given that, a priori, there is no physical or statistical reason to choose one metric over the others, we chose one or more examples from each family and then evaluated their performance when applied to our data. The histogram metrics are discussed in the Appendix in more detail. After initial evaluation, we chose a single metric to present the final analysis, based on how the distance metric separated outlier data sets relative to “normal” data. For this analysis, all histograms have been normalized using the total number of binned values. The rationale for investigating a broad range of metrics outside the simplest Minkowski family is that other families address the insensitivity of the Minkowski metrics to simple offsets between histograms and we expect that the protocol parameters investigated here will create subtle changes in histogram shape as well as potential offsets.

### Previous use of histograms in DWI

Various histogram analyses have been performed on derived data maps in MRI-based diffusion experiments. Published studies however, have reported only the mean, median, peak height and, less often, quartiles. In some cases, changes in those quantities were measured after some intervention. Here we briefly list several examples that span the range of common analyses. Bester et al. (2015) compared the mean values of mean kurtosis (MK), MD, and FA histograms between MS patients and controls. Kang et al. (2011), calculated differences in apparent diffusion coefficient (ADC) histogram percentile values of tumor volume data in an effort to determine glioma grade and evaluate the diagnostic performance of ADC maps at two different *b*-values. Pope et al. (2011, 2012) has fit the post-treatment ADC histogram to a bimodal distribution and analyzed the mean ADC for each component. Nusbaum et al. (2000) created ADC whole-brain histograms of different categories of MS patients and reported the increase in mean ADC_av_ of controls and each different category using changes in peak location and peak height. Rovaris et al. (2003) used data from normal-aging subjects to create whole brain histograms of ADC and FA, analyzing both the mean value and peak height vs. age. Several studies have performed more involved analysis of histogram data. For example, Yankeelov et al. (2007) acquired dynamic contrast enhancement and ADC maps in a study of breast cancer, created ADC histograms and looked for statistically significant changes in individual bin frequency between pre- and post-treatment histograms. Goodyear et al. (2015) studied changes in optic nerve mean MD, FA, axial (AD), and radial diffusivity (RD) as well as changes in histogram skewness of those quantities in optic neuritis patients. Wagner et al. (2016) characterized pediatric cerebellar tumors using the 25th percentile, 75th percentile, and skewness of FA, MD, AD), and RD. Steffen-Smith et al. (2014) analyzed MD histogram through the standard deviation, skewness, measures of histogram asymmetry: length of left tail > right tail (negative skewness) or length of right tail > left tail (positive skewness), peak location (mode), and peak height. MD values were also fitted using a two-normal mixture distribution model. Finally, Tozer et al. (2006) used principal-component and linear-discriminant analysis on T1 and ADC histograms from multiple sclerosis patients and compared metrics of these analyses to the standard peak height and location metrics.

## Materials and methods

### Data preparation and processing

This study was approved by the Partners Human Research Committee. Subjects have granted their written informed consent for the use of this data.

Here we provide details on how the data set was constructed using a database of radiology reports from hospitals within the Partners HealthCare Network. The code used in the selection and analysis of the data can be found on GitHub at https://github.com/gcwarner1/DTIDistance. The reports were first sorted to find patients who underwent brain MRI scans, but who were ultimately free of any pathology. We include information on the sorting process to show the steps necessary to construct such a data set from a clinical data repository and to contextualize the data set that was finally analyzed in this study.

We obtained DTI scans of individuals with normal brain morphology from the Partners Research Patient Data Registry (RPDR) (Nalichowski et al., 2006) clinical database via a two-fold query by age range and diagnosis. The patient age range was 18–54 and the diagnosis selection terms were: “Chronic migraine without aura,” “Migraine,” “Migraine with aura,” “Migraine without aura,” “Migraine unspecified,” “Ophthalomplegic migraine,” “Periodic headache syndromes in child or adult,” or “Persistent migraine aura without cerebral infarction.” From the results of this query we selected all reports that were tagged: “BRAIN MRI,” “MR Brain w/o Contrast,” “MRD BRAIN,” “MRI BRAIN,” “MRI SCAN BRAIN,” “MRI Brain WITHOUT,” “MRI Brain W/O Con MRI Brain W/O Con,” or “MRI BRAIN WO CONT.” This query yielded 232,922 radiology reports, each of which described a single clinical visit.

To find the scan sessions that included diffusion imaging, we then selected only those reports that contained the terms “DTI” and/or “diffusion,” which yielded 3,035 reports. We then used natural language processing (in-house python code) to remove all reports mentioning artifacts or morphological abnormalities. This filtering was performed in two steps. First, we removed all reports containing any of the terms: “braces,” “resection,” “craniotomy,” “callosotomy,” “cingulotomy,” “lobotomy,” “hemicraniotomy,” or “lobectomy.” Second, we removed all reports that contained any of the terms: “ischemia,” “tumor,” “stroke,” “ischemic,” “neurofibromatosis,” “infarct,” “glioblastoma,” “artifact,” “artifactual,” “mass effect,” “hyperintense,” or “hyper intensity” that did not also include any of the terms: “no,” “none,” “negative,” “not,” “without,” “inconsistent,” “normal,” “performed,” “obtained,” “acquired,” or “unremarkable” in the same sentence. Constructing this list was an iterative process that involved examining a large random sampling of the reports to determine commonly used terms.

Together, these two steps reduced the number of qualifying radiology reports to 1,438. All MRI data from these remaining reports was then downloaded from the Partners database. This yielded 26,013 DICOM series (note that these series included all of the scans from a particular scan session, including non-diffusion scans), all of which were either JPEG or RLE compressed. These series were decompressed using the software packages dcmdjpeg (OFFIS, Oldenburg, Germany) and dcmdrle (OFFIS, Oldenburg, Germany), respectively. Thirty-four of the series were corrupt and failed to decompress resulting in 25,979 decompressed series. All data whose sequence, *b*-value, and protocol DICOM tags (including vendor specific private tags known to contain diffusion information) did not contain any of the strings: “tof,” “fl3d,” “memp,” “fse,” “grass,” “3-Plane,” or “gre,” but did contain at least one of the strings: “ep2,” “b,” “ep_,” “1000,” “directional,” or “dif” were considered to be diffusion-related data and all others were removed, reducing the number of series to 7,521. This set of tag strings was determined by examining a listing of DICOM tag values for the following tags:

Pulse Sequence: (0018,0024) (0018,0020)
Protocol: (0018,1030)
*B*-Value: (0018,9087)
Siemens Private *B*-Value: (0019,100C)
GE Private Sequence: (0019,109C)
GE Private *B*-Value: (0043,1039)

The remaining volumes were then converted to NIfTI format using dcm2nii (https://github.com/rordenlab). Of these 7,521 series, 6,212 were scanner-calculated diffusion metric maps (e.g., ADC, FA) rather than series of diffusion-weighted images. These maps were not used in this study to ensure that each analyzed map was processed using the same pipeline. This step resulted in 1,309 NIfTI files and their corresponding *b*-value and b-vector files. Next, we removed all data which did not contain both *b*-value and b-vector files, along with all data containing less than six non-zero *b*-values, and data for which the number of *b*-values did not match the number of gradient directions. This filtration step resulted in 1,266 usable data sets.

We then eddy- and motion-corrected each volume within each diffusion data set using FSL's (Jenkinson et al., 2012) (FMRIB, Oxford, UK) eddy_correct tool and the gradient direction vectors were corrected for the observed motion. Calculation of diffusion metrics was then performed using FSL's dtifit tool. At this stage, we also removed data with additional issues. Fourteen data sets had gradient direction vectors that contained all zeros and a further data set contained a negative *b*-value. In addition, we set an upper threshold of 1.0 for the FA maps thereby removing non-physical values. We also removed the corresponding voxel in the MD map for each FA map voxels found to be above 1.0.

### Histogram generation for diffusion and distance metrics

**Figure 1:**
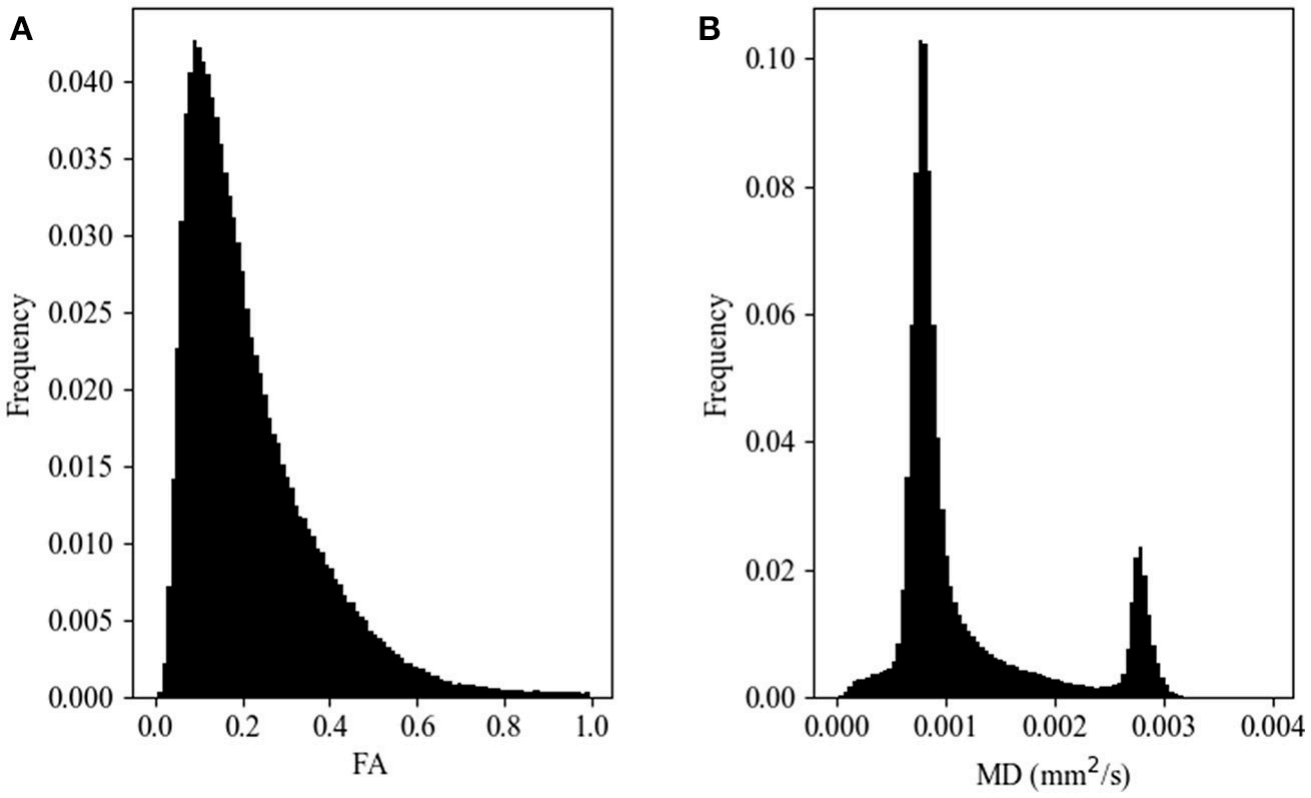
Representative histograms of diffusion metric map values for fractional anisotropy, FA **(A)** and mean diffusivity, MD **(B)** maps. These data arise from a single subject scanned on a 1.5T GE scanner at a *b*-value of 1,000 s/mm^2^ and 25 gradient directions.

Histograms of FA and MD values were constructed for each of the remaining data sets. Each histogram was calculated using 100 bins, with ranges (0.0–1.0] for FA and (0.0–0.004] mm^2^/s for MD. The *b*-values, number of gradient directions, scanner vendor, and field strength were recorded for each volume/histogram. These tags were used to construct data sets with identical parameters used in the comparisons described below. Sample FA and MD histograms are shown in Figure 1. Note that in neither case are the histograms Gaussian.

The general methodology we followed was to first create subsets of diffusion metric (FA and MD) histograms based on vendor, field strength, *b*-value, and number of gradient directions, then calculate histogram distance metrics between all pairs of histograms within that subset. This calculation resulted in a set of distance metrics. This set of distance metric values was then formed into histograms and these distance metric histograms were then compared to that calculated from other related subsets using statistical tests described below. Because the data used in this study arose from a clinical repository and were not acquired using a harmonized protocol, the ability to make meaningful comparisons depended upon the number of data sets with a given set of acquisition parameters in the repository. Shown in Table 1 are the relevant acquisition parameters for the available data. As an example, note that the majority of GE data is at a field strength of 1.5T and a *b*-value of 1,000 s/mm^2^ while the Siemens data is more evenly divided between 1.5T and 3.0T data and there is a range of *b*-values. Therefore, exactly matching all of the acquisition parameters is impossible for some obvious data subsets, but the process does serve to illustrate the situation faced when trying to assemble a data set for reuse from a repository containing data acquired with many different protocols.

**Table 1:** Composition of the 1,266 data sets used for analysis broken down by vendor and static field strength and *b*-value (rows), as well as the number of gradient directions (columns).

**Field Strength**	***b*-value**	**Number Grad Directions**																	
		**6**	**8**	**9**	**12**	**14**	**15**	**16**	**17**	**24**	**25**	**29**	**30**	**31**	**35**	**36**	**48**	**58**	**60**
**SIEMENS**																			
1.5T	700	0	0	0	0	0	0	0	0	0	0	0	0	0	0	0	0	0	0
	1,000	0	0	0	0	0	0	0	0	3	0	0	52	0	1	89	1	8	0
3.0T	700	0	0	0	0	0	0	0	0	0	0	0	2	0	0	1	0	0	37
	1,000	0	0	1	24	0	0	1	0	64	1	0	32	0	0	0	0	0	3
**GE**																			
1.5T	700	0	0	0	0	0	0	0	0	0	0	0	0	0	0	0	0	0	0
	1,000	269	1	0	0	1	30	0	9	0	560	15	0	21	1	0	0	0	0
3.0T	700	0	0	0	0	0	0	0	0	0	0	0	0	0	0	0	0	0	0
	1,000	0	0	0	0	0	0	0	0	0	32	0	0	0	0	0	0	0	0

*The numbers indicate the number of data sets for that combination of manufacturer, field strength, b-value and number of gradient directions (e.g., there were 32 GE data sets with a field strength of 3.0T, a b-value of 1,000 s/mm^2^, and 25 gradient directions). There are 6 data sets not represented in this table. Five data sets had multiple b-values, ranging from 2 to 21 b-values. The other data set had a single b-value that was not either of the two listed in the table above. All were from Siemens scanners with a static field strength of 3.0T. These six data sets were only included in comparisons in which b-value was not a controlled variable (e.g., all GE vs. all Siemens)*.

The specific comparisons investigated here were selected as those that had sufficient number of data sets to make the comparisons meaningful. The following comparisons were performed (if a specific tag value is not noted, no restriction is set on its value):

1. Within vendor (Siemens, GE)
2. Between vendor (Siemens vs. GE)
3. Between vendor, field strength of 1.5T.
4. Between vendor, *b* = 1,000 s/mm^2^
5. Within Siemens, *b* = 1,000 s/mm^2^ vs. *b* = 700 s/mm^2^
6. Within Siemens, field strength (1.5T vs. 3.0T)
7. Between vendor, *b* = 1,000 s/mm^2^, 30 gradient directions, 1.5T
8. 1.5T vs. 3.0T Siemens, *b* = 1,000 s/mm^2^, 30 gradient directions,.
9. Within GE, *b* = 1,000 s/mm^2^, 1.5T, 6 vs. 25 gradient directions

All distance metric histograms were then normalized such that the integral of the histogram was equal to one. One goal of the project was to determine the shape and range of the histogram-distance metric histograms, as these could be used as normative distributions against which the similarity of newly acquired data could be determined. Another goal was to compare the shape and extent of the distance metric histograms for the above comparisons to see the effect of the different acquisition parameter choices on the similarity of the data.

### Selection of distance metric

The histogram distance calculations in this work were performed with each of the 13 distance metrics. The details of these metrics are given in the Appendix. Some metrics performed better than others and we selected a single metric for presentation of the results. Based on the dynamic range of the metric and the distance calculated for data sets with visible artifacts, we chose the Hellinger metric for presentation.

### Use of histogram distance to discover volumes with severe artifacts

Initially, each distance metric histogram for each comparison was used to identify data sets that contained artifacts. Several of the FA distance metric histograms appeared to be bimodal with a second, smaller peak appearing in the (right) tail of the primary distribution. These peaks were most pronounced in the Canberra, City-Block, Euclidean, Intersection, and Hellinger metrics. Of the 884 individual data sets represented in the smaller peak of the all-GE vs. all-Siemens comparison, three data sets appeared in more than two comparisons, signaling that these data sets were dissimilar to most of the other data sets. These three sets appeared in 570, 309, and 168 comparisons respectively. Visual inspection of these three data files revealed significant artifacts. These three FA volumes were removed from the FA data set and the histograms were regenerated. This new set of histograms again contained an outlier peak. Two data sets were disproportionately represented in this outlier mode occurring 299 and 298 times respectively while no other data set occurred more than twice. Visual inspection revealed improper slice arrangement and other artifacts in these files. They were both removed from our data set and the histograms were regenerated, this time, yielding unimodal, though decidedly non-normal, histograms. For each FA map histogram removed in this process, the corresponding MD map histogram was also removed.

This process was then repeated for the MD maps where we discovered a data set in which the subject was scanned with a head-and-neck coil and the field-of-view contained a larger portion of the spinal cord than did the other data sets. This MD volume and the corresponding FA volume were then removed, and the histogram distance metric histograms were recalculated. Note that, while we were also able to identify diffusion metric maps that had elevated noise using this process, these were not removed from consideration; only maps with clear artifacts were removed.

### Comparison between histogram distance distributions

As the histogram distance histograms were non-Gaussian and there was no “ideal” histogram to compare them to, we used two different non-parametric methods to determine the similarity between histogram-distance metric histograms for each chosen comparison. First, we used the Mann–Whitney *U*-test in which values from both distributions are chosen and ranked by value. This method tests the similarity of two distributions through a null hypothesis that a randomly-chosen sample from the first distribution will be less than or equal to a randomly-selected sample from the second distribution. The Mann–Whitney *U*-test therefore, tests for global similarity. Second, we used the two-sample Kolmogorov–Smirnov test, which compares bin values of the cumulative histograms of the two distributions and is therefore sensitive to the range, location and shape of the histograms.

To better visualize the distributions, we converted the histograms into box-and-whisker plots with the whiskers extending 1.5 times the interquartile range past the third quartile. We used these values for the whiskers rather than the fourth quartile so that noisy, but valid, data sets would not extend the whiskers into a region where there are far fewer values and create what we believe would have been an unrepresentative view of the data. The distance between the median and 1.5 times the interquartile range past the third quartile was used to describe the variance in the data in the calculations described below. All data points, including those outside the whiskers of the box-and-whisker plots, were included in the Mann–Whitney *U*-test. The tests were performed for each of the nine comparisons listed above.

In addition, we also created cumulative histogram plots of the histogram distance metrics for each subset of data. Using these cumulative histograms, we calculated the Kolmogorov–Smirnov statistic in each case. This presentation allows the visualization of shape differences between the two compared histograms while the box-and-whisker plots are better for visualizing the median and range of the histograms.

## Results

As discussed above, the goal of this work is to show the usefulness of the histogram distance in characterizing the similarity of data, particularly retrospective data from a repository. Unless the imaging protocols were harmonized or only a single protocol is available on a single scanner, repository data will have been acquired with varying protocols and it is often not clear the magnitude of the effect that the parameter variations have on the similarity of the data. The approach we take is to present results from the general to the specific, i.e., to present results for data that have the least acquisition parameters in common to the most. For example, how similar are data from different vendors, irrespective of other acquisition parameters (least specific)? Or how similar are data that are matched in field strength, *b*-value, and number of gradient directions, but not vendor (most specific)? Through these comparisons, one can gain an understanding of the relative importance various parameters.

The set of histogram distance metrics investigated here generally performed similarly, but differed slightly in their ability to separate data sets that produced distances in the tails on either side of the distribution. Of the 13 metrics tested, the Hellinger metric was chosen as being representative of those metrics that met the criteria discussed above. We therefore use that metric to present results for differences due to scanner manufacturer, *b*-value, and field strength, all of which proved to have a significant influence over the shape of the distance metric histograms. In what follows, “outliers” will be defined as 1.5 times the interquartile range past the third quartile.

### Comparison between manufacturers

**Figure 2:**
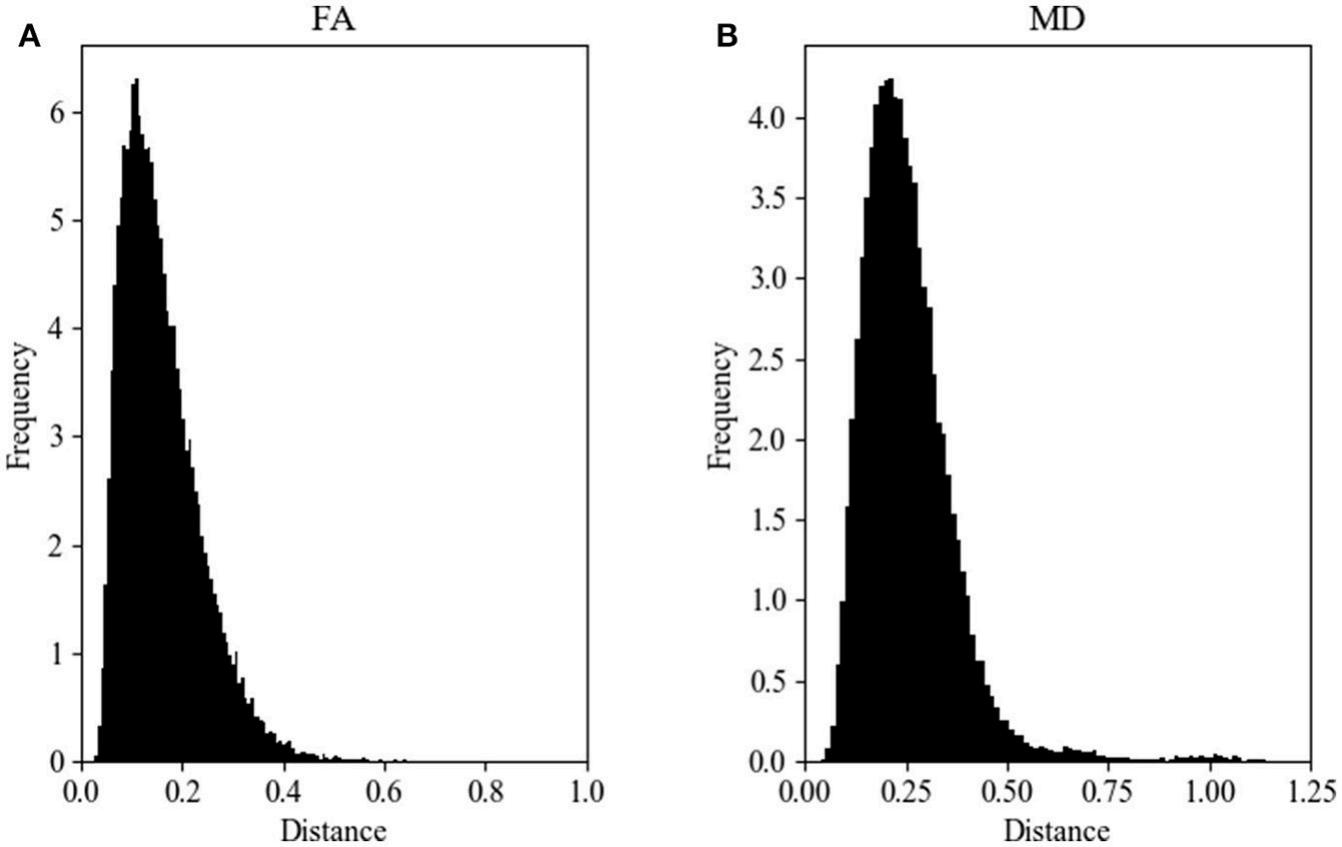
FA **(A)** and MD **(B)** map Hellinger histogram distance metric for the within-group Siemens (all *b*-values, all gradient directions, all field strengths) (number of comparisons = 47,586).

**Figure 3:**
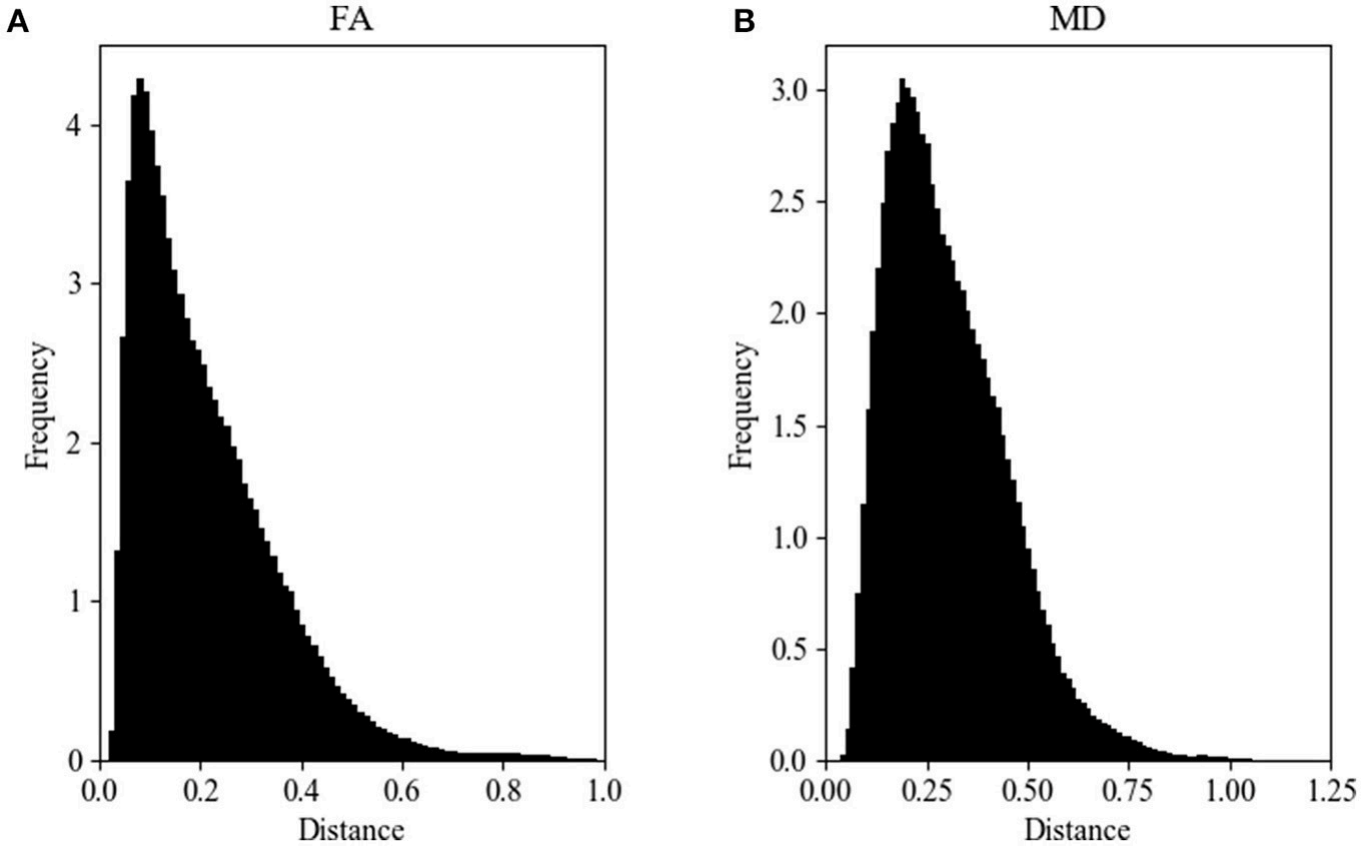
FA **(A)** and MD **(B)** map Hellinger histogram distance metric for the within-group GE (all *b*-values, all gradient directions, all field strengths) (number of comparisons = 434,778).

Figure 2A shows the Hellinger distance metric values between the FA maps of data collected on Siemens scanners (*n* = 47,586 comparisons, all scans). Figure 3A shows the Hellinger distance metric values between the FA maps of data collected on GE scanners (*n* = 434,778 total comparisons, all scans). The difference between the two distributions was statistically significant according to the Mann–Whitney U (*U* = 8.5e9, *p* = 4e-126) and the Kolmogorov–Smirnov (K–S = 0.2605, *p* = 9e-1700) tests. The Hellinger distance between the median and the upper limit, excluding outliers, was 0.3899 for the GE data and 0.2010 for Siemens data.

Figure 2B shows the Hellinger distance metric values between the MD maps of data collected on Siemens scanners (*n* = 47,586 comparisons, all scans). Figure 3B shows the Hellinger distance metric values between the MD maps of data collected on GE scanners (*n* = 434,778 total comparisons, all scans). The difference between the two distributions was statistically significant according to the Mann–Whitney U (*U* = 8.5e9, *p* = 2e-130) and the Kolmogorov–Smirnov (K–S = 0.2011, *p* = 3e-170) tests.

**Figure 4:**
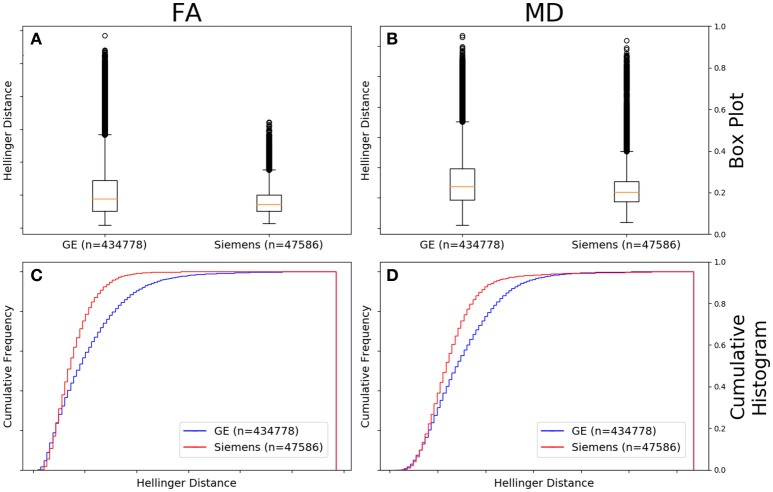
FA **(A)** and MD **(B)** map box-and-whisker plot of the histogram distance values for all GE (number of comparisons = 434,778) vs. all Siemens (number of comparisons = 47,586). Cumulative histograms for these data are shown for FA **(C)** and MD **(D)**.

Figure 4 shows the data of Figures 2, 3 in the form of a whisker plot. The whisker extends 1.5 times the interquartile range past the third quartile with the “outlier” distances plotted as individual points. The Hellinger distance between the median and the upper limit, excluding outliers, was 0.4277 for the GE data and 0.2705 for Siemens data.

### Comparison between manufacturers; *b*-value controlled

**Figure 5:**
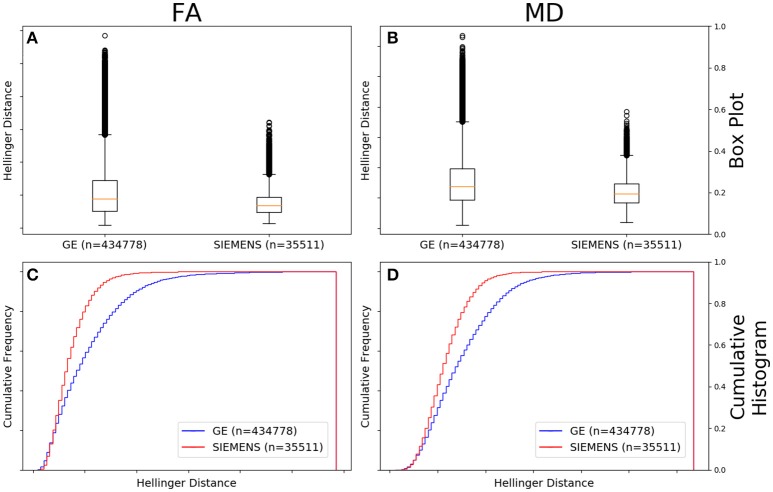
FA **(A)** and MD **(B)** map box-and-whisker plot for GE (number of comparisons = 434,778) vs. Siemens (number of comparisons = 35,511) histogram distances for data collected with a *b*-value of 1,000 s/mm^2^. Cumulative histograms for these data are shown for FA **(C)** and MD **(D)**.

Figure 5A shows that the FA maps of data collected on a GE scanner with a *b*-value of 1,000 s/mm^2^ (*n* = 434,778 comparisons) had a wider range of Hellinger distance distributions compared to Siemens data with a *b*-value of 1,000 s/mm^2^ (*n* = 35,511 comparisons). Figure 5C shows the cumulative frequency plots for each FA map Hellinger distance distribution. The two distributions differed significantly according to the Mann–Whitney U (*U* = 5.9e9, *p* = 8e-126) and the Kolmogorov–Smirnov (K–S = 0.2378, *p* = 5e-170) tests. The Hellinger distance between the median and the upper limit, excluding outliers, was 0.3899 for the GE 1,000 s/mm^2^ *b*-value data and 0.1916 for the Siemens 1,000 *b*-value data.

Figure 5B shows that the MD maps of data collected on a GE scanner with a *b*-value of 1,000 (*n* = 434,778 comparisons) had a wider range of Hellinger distance distributions compared to Siemens data with a *b*-value of 1,000 s/mm^2^ (*n* = 35,511 comparisons). Figure 5D shows the cumulative frequency plots for each MD map Hellinger distance distribution. The two distributions differed significantly according to the Mann–Whitney U (*U* = 5.8e9, *p* = 4e-130) and the Kolmogorov–Smirnov (K–S = 0.2193, *p* = 7e-174) tests. The Hellinger distance between the median and the upper limit, excluding outliers, was 0.4277 for the GE *b* = 1,000 s/mm^2^ data and 0.2543 for the Siemens *b* = 1,000 s/mm^2^ data.

### Comparison between *b*-values; vendor controlled

**Figure 6:**
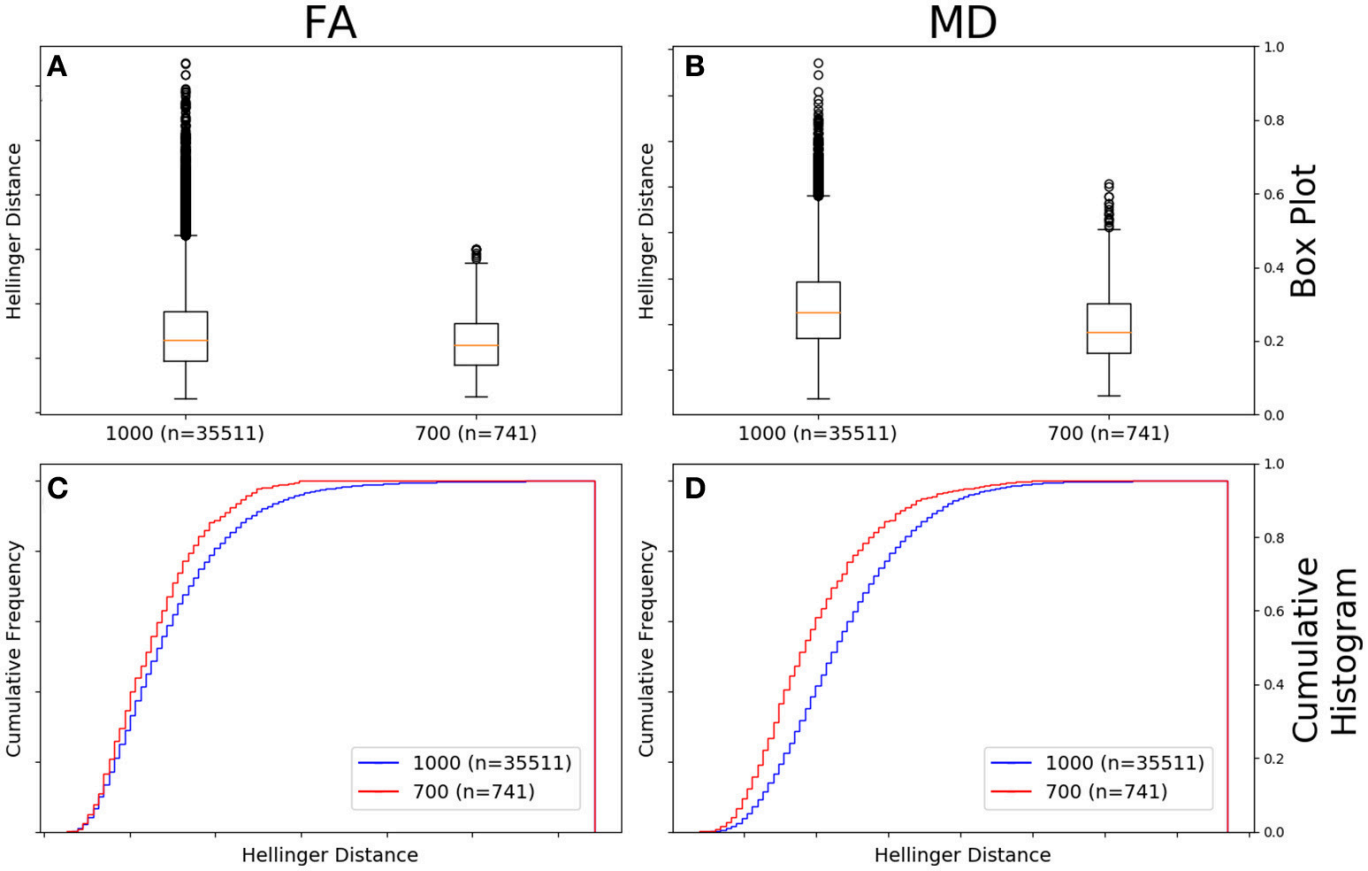
FA **(A)** and MD **(B)** map box-and-whisker plot for Siemens *b* = 1,000 s/mm^2^ (number of comparisons = 35,511) vs. Siemens *b* = 700 s/mm^2^ (number of comparisons = 741) data. Cumulative histograms for these data are shown for FA **(C)** and MD **(D)**.

Figure 6A shows there was a significant difference between the Hellinger distances distributions of the FA maps of Siemens data with a *b*-value of 700 s/mm^2^ (*n* = 741 comparisons) and Siemens data with a *b*-value of 1,000 s/mm^2^ (*n* = 35,511 comparisons) according to the Mann–Whitney U (*U* = 8.5e9, *p* = 4e-80) and the Kolmogorov–Smirnov (K–S = 0.1001, *p* = 8e-7) tests. The Hellinger distance between the median and upper limit, excluding outliers, was 0.1916 for the Siemens data with a *b*-value of 1,000 s/mm^2^ and 0.1516 for the Siemens data with a *b*-value of 700 s/mm^2^. Figure 6C shows the cumulative frequency plots for each FA map Hellinger distance distribution.

Figure 6B shows there was a significant difference between the Hellinger distances distributions of the MD maps of Siemens data with a *b*-value of 700 s/mm^2^ (*n* = 741 comparisons) and Siemens data with a *b*-value of 1,000 s/mm^2^ (*n* = 35,511 comparisons) according to the Mann–Whitney U (*U* = 8.5e9, *p* = 2e-101) and the Kolmogorov–Smirnov test (K–S = 0.2005, *p* = 5e-26). The Hellinger distance between the median and upper limit, excluding outliers, was 0.2543 for the Siemens data with a *b*-value of 1,000 s/mm^2^ and 0.2244 for the Siemens data with a *b*-value of 700 s/mm^2^. Figure 6D shows the cumulative frequency plots for each MD map Hellinger distance distribution.

### Comparison between field strengths; vendor controlled

**Figure 7:**
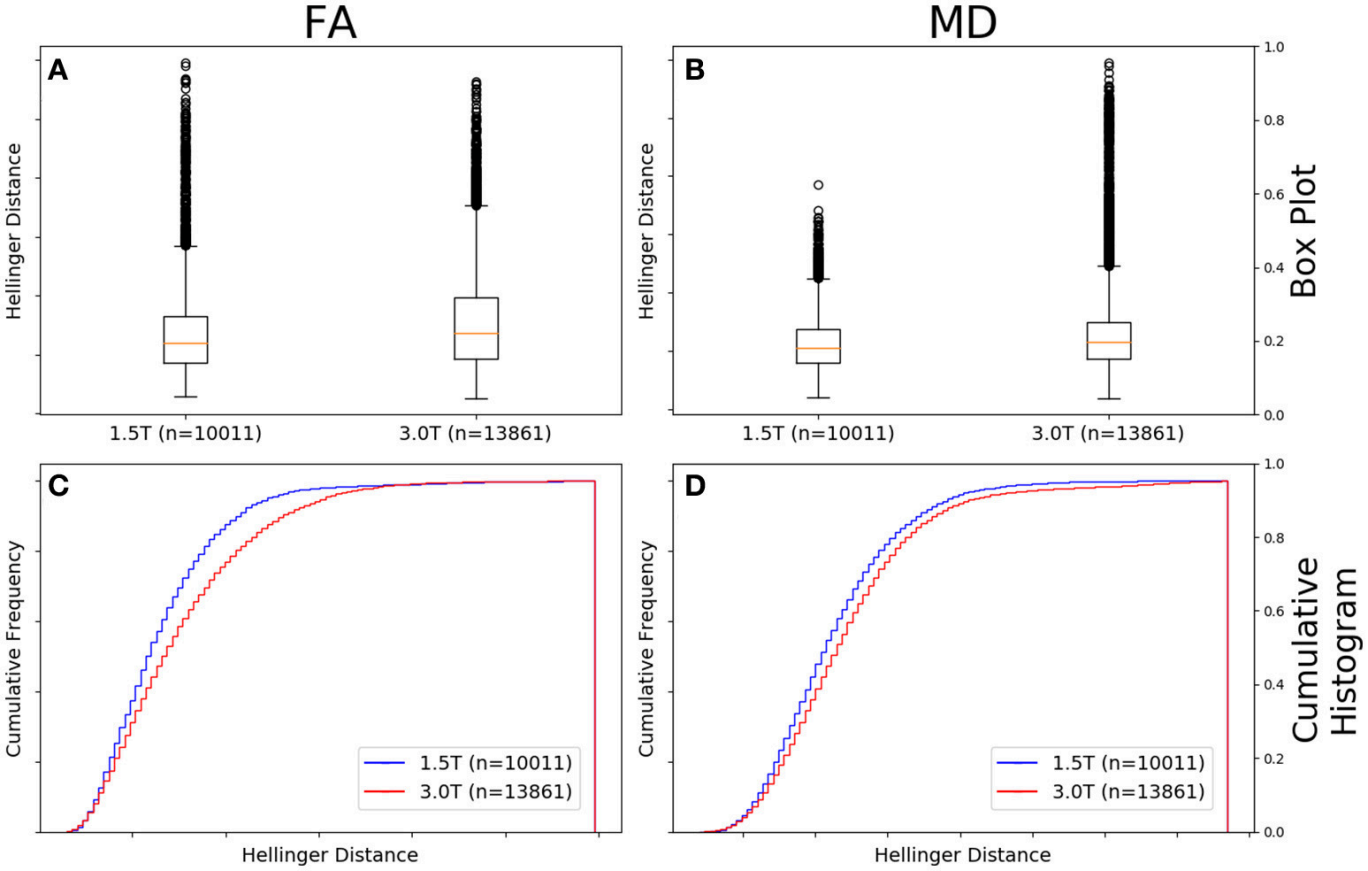
FA **(A)** and MD **(B)** map box-and-whisker plot for Siemens 1.5T (number of comparisons = 10,011) vs. Siemens 3.0T (number of comparisons = 13,861). Cumulative histograms for these data are shown for FA **(C)** and MD **(D)**.

Figure 7A shows there was a significant difference between the Hellinger distance distributions of the FA maps of Siemens data with a static field strength of 3.0T (*n* = 13,861 comparisons) and Siemens data with a static field strength of 1.5T (*n* = 10,011 comparisons) according to the Mann–Whitney U (*U* = 6.0e7, *p* = 3e-77) and the Kolmogorov–Smirnov test (K–S = 0.1178, *p* = 1e-70) tests. The Hellinger distance between the median and upper limit, excluding outliers, was 0.2168 for the 3.0T group and was 0.1641 for the 1.5T group. Figure 7C shows the cumulative frequency plots for each FA map Hellinger distance distribution.

Figure 7B shows there was a significant difference between the Hellinger distance distributions of the MD maps of Siemens data with a static field strength of 3.0T (*n* = 13,861 comparisons) and Siemens data with a static field strength of 1.5T (*n* = 10,011 comparisons) according to the Mann–Whitney U (*U* = 6.2e7, *p* = 1e-45) and the Kolmogorov–Smirnov test (K–S = 0.0813, *p* = 7e-34) tests. The Hellinger distance between the median and upper limit, excluding outliers, was 0.2610 for the 3.0T group and was 0.2379 for the 1.5T group. Figure 7D shows the cumulative frequency plots for each MD map Hellinger distance distribution.

### Comparison between field strengths; *b*-value, vendor, and number of gradient directions controlled

**Figure 8:**
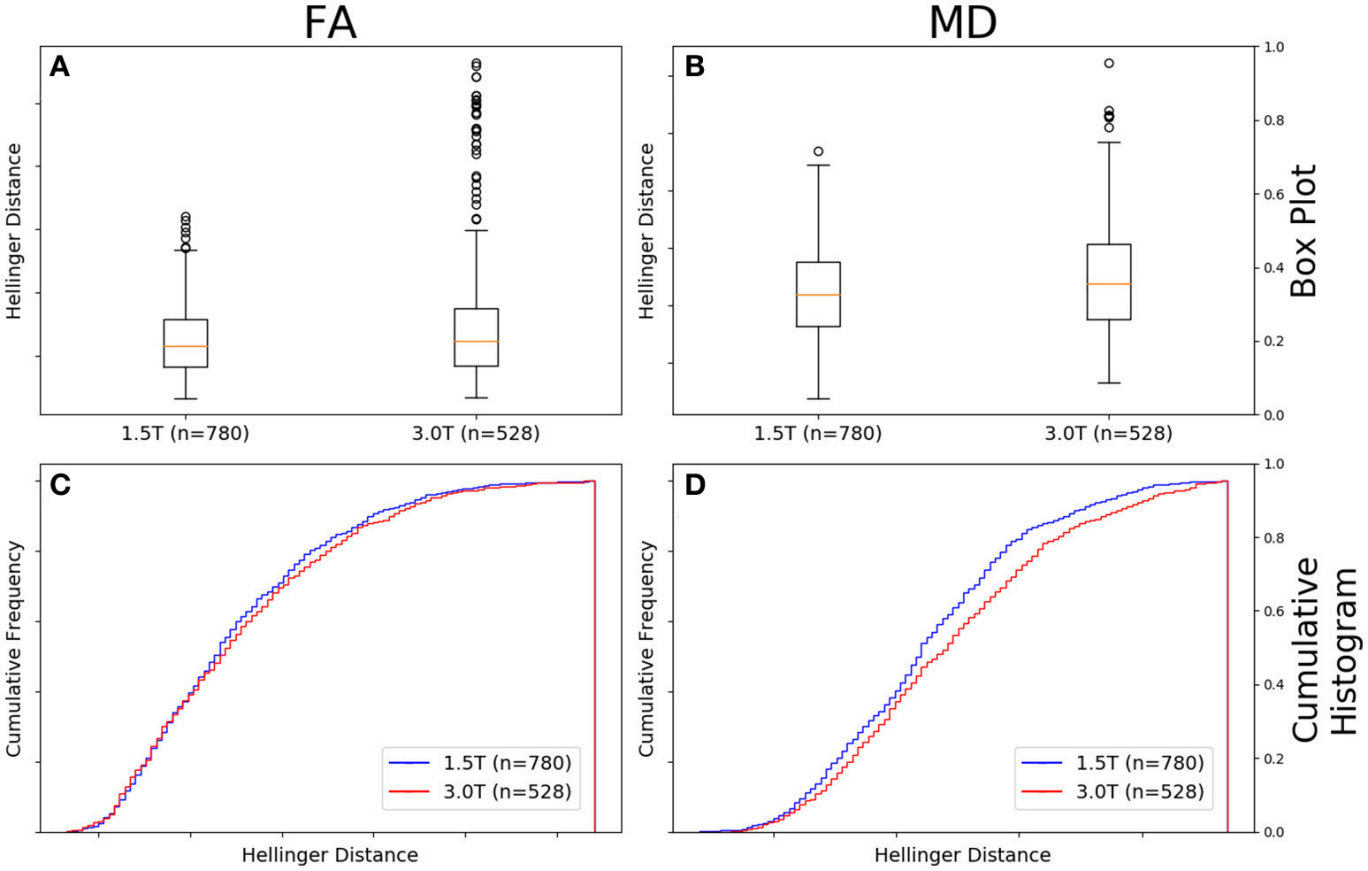
FA **(A)** and MD **(B)** map box-and-whisker plot Siemens 1.5T (number of comparisons = 780) vs. Siemens 3.0T (number of comparisons = 528) for *b* = 1,000 s/mm^2^ and 30 gradient directions. Cumulative histograms for these data are shown for FA **(C)** and MD **(D)**.

Figure 8A shows there was a difference between the Hellinger distance distributions of the FA maps of Siemens data with a *b*-value of 1,000 s/mm^2^, 30 gradient directions, and a static field strength of 3.0T (*n* = 528 comparisons) and Siemens data with a *b*-value of 1000 s/mm^2^, 30 gradient directions, and a static field strength of 1.5T (*n* = 780 comparisons) according to the Mann–Whitney U (*U* = 1.9e5, *p* = 9e-3 and the Kolmogorov–Smirnov test (K–S = 0.0847, *p* = 0.02046) tests. The Hellinger distance between the median and upper limit, excluding outliers, was 0.1516 for the 1.5T group and was 0.1767 for the 3.0T group. Figure 8C shows the cumulative frequency plots for each FA map Hellinger distance distribution.

Figure 8B shows there was a difference between the Hellinger distance distributions of the MD maps of Siemens data with a *b*-value of 1,000 s/mm^2^, 30 gradient directions, and a static field strength of 3.0T (*n* = 528 comparisons) and Siemens data with a *b*-value of 1,000 s/mm^2^, 30 gradient directions, and a static field strength of 1.5T (*n* = 780 comparisons) according to the Mann–Whitney U (*U* = 180,084.0, *p* = 6e-05) and the Kolmogorov–Smirnov test (K–S = 0.1196, *p* = 0.000214) tests. The Hellinger distance between the median and upper limit, excluding outliers, was 0.2243 for the 1.5T group and was 0.2243 for the 3.0T group. Figure 8D shows the cumulative frequency plots for each MD map Hellinger distance distribution.

### Comparison between number of gradient directions; *b*-value, vendor, and field strength controlled

**Figure 9:**
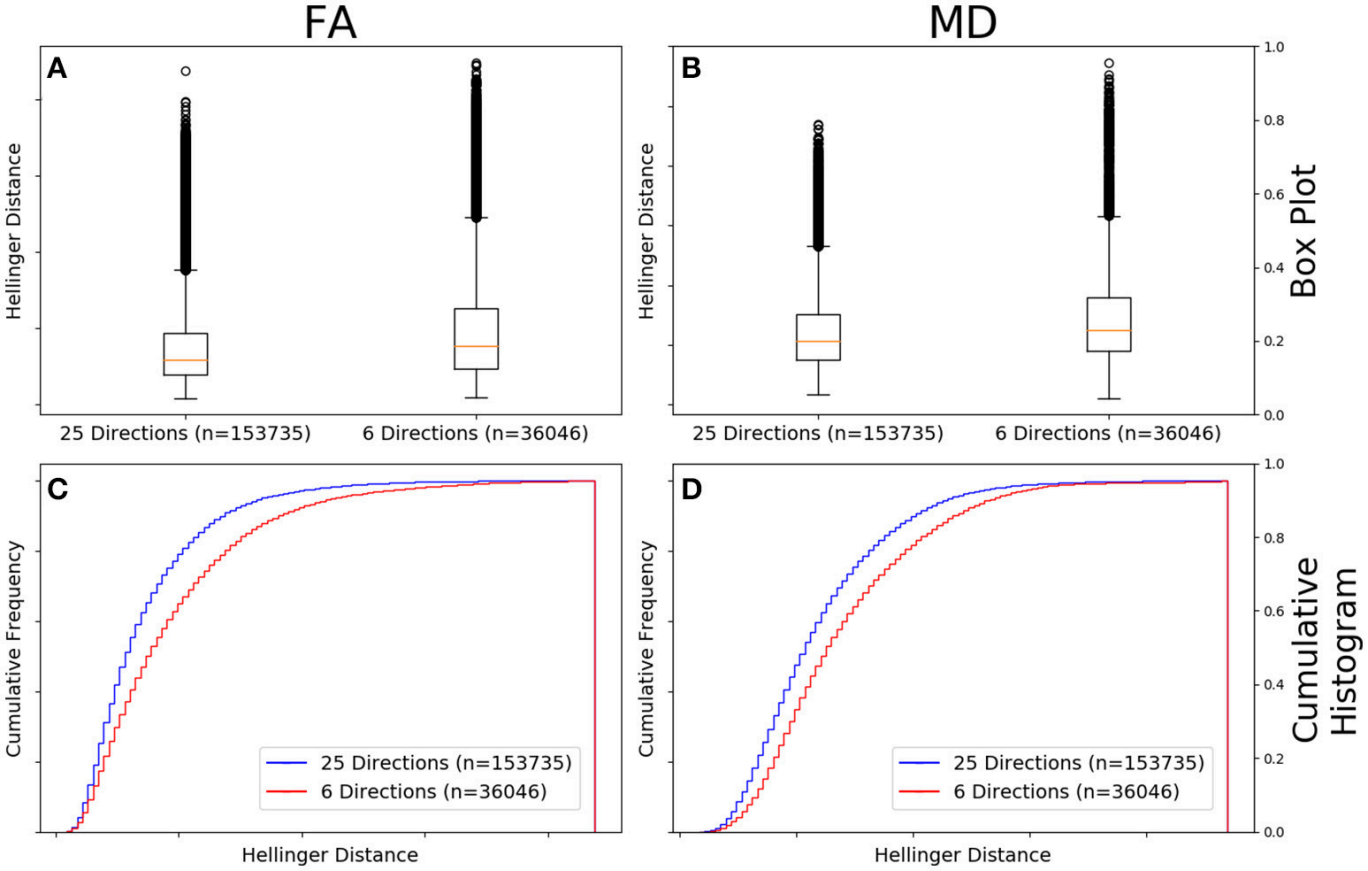
FA **(A)** and MD **(B)** map box-and-whisker plot for GE 6 gradient directions (*n* = 36,046 comparisons) vs. GE 25 gradient directions [(*n* = 153,735 comparisons) for b = 1,000 s/mm^2^ and 1.5T. Cumulative histograms for these data are shown for FA **(C)** and MD **(D)**].

Figure 9A shows that the FA maps of GE data with a *b*-value of 1,000 s/mm^2^, a static field strength of 1.5T, and 25 gradient directions (*n* = 153,735 comparisons) had a narrower range of Hellinger distance distributions compared to GE data with a *b*-value of 1,000 s/mm^2^, a static field strength of 1.5T, and 6 gradient directions (*n* = 36,046 comparisons). Figure 9C shows the cumulative frequency plots for each FA map Hellinger distance distribution. The two distributions differed significantly according to both the Mann–Whitney *U*-test (*U* = 2.2e9, *p* = 1e-130) and the Kolmogorov–Smirnov test (K–S = 0.1555, *p* = 2e-174). The Hellinger distance between the median and upper limit, excluding outliers, was 0.2358 for the 25 gradient directions group and was 0.3355 for the 6 gradient directions group.

Figure 9B the MD maps of GE data with a *b*-value of 1,000 s/mm^2^, a static field strength of 1.5T, and 25 gradient directions (*n* = 153,735 comparisons) had a narrower range of Hellinger distance distributions compared to GE data with a *b*-value of 1,000 s/mm^2^, a static field strength of 1.5T, and 6 gradient directions (*n* = 36,046 comparisons). Figure 9D shows the cumulative frequency plots for each MD map Hellinger distance distribution. The two distributions differed significantly according to both the Mann–Whitney *U*-test (*U* = 2.2e9, *p* = 9e-130) and the Kolmogorov–Smirnov test (K–S = 0.1281, *p* = 3e-173). The Hellinger distance between the median and upper limit, excluding outliers, was 0.3198 for the 25 gradient directions group and was 0.3816 for the 6 gradient directions group.

### Comparison between vendors; number of gradient directions, field strength, and *b*-value controlled

**Figure 10:**
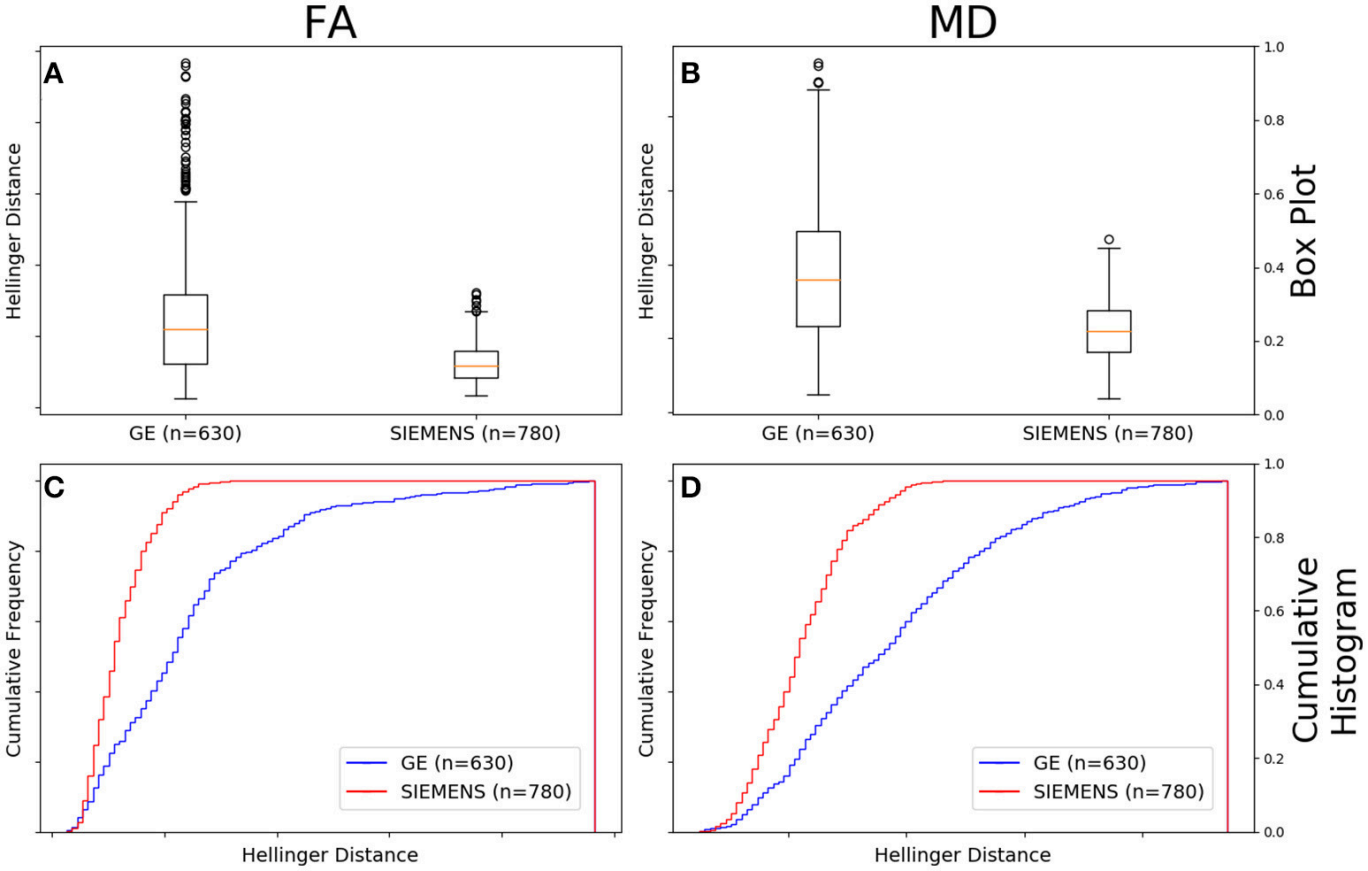
FA **(A)** and MD **(B)** map box-and-whisker plot for GE (number of comparisons = 630) vs. Siemens (number of comparisons = 780) for 30 gradient directions, *b* = 1,000 s/mm^2^ and 1.5T. Cumulative histograms for these data are shown for FA **(C)** and MD **(D)**.

Figure 10A shows that the FA maps of GE data with a *b*-value of 1,000 s/mm^2^, a static field strength of 1.5T, and 30 gradient directions (*n* = 630 comparisons) had a wider range of Hellinger distance distributions compared to Siemens data with a *b*-value of 1,000 s/mm^2^, a static field strength of 1.5T, and 30 gradient directions (*n* = 780 comparisons). Figure 10C shows the cumulative frequency plots for each FA map Hellinger distance distribution. The two distributions differed significantly according to both the Mann–Whitney *U*-test (*U* = 1.2e5, *p* = 9e-61) and the Kolmogorov–Smirnov test (K–S = 0.4649, *p* = 1e-66). The Hellinger distance between the median and upper limit, excluding outliers, was 0.3604 for the GE group and was 0.1526 for the Siemens group.

Figure 10B shows that the MD maps of GE data with a *b*-value of 1,000 s/mm^2^, a static field strength of 1.5T, and 30 gradient directions (*n* = 630 comparisons) had a wider range of Hellinger distance distributions compared to Siemens data with a *b*-value of 1,000 s/mm^2^, a static field strength of 1.5T, and 30 gradient directions (*n* = 780 comparisons). Figure 10D shows the cumulative frequency plots for each MD map Hellinger distance distribution. The two distributions differed significantly according to both the Mann–Whitney *U*-test (*U* = 1.2e5, *p* = 7e-65) and the Kolmogorov–Smirnov test (K–S = 0.4482, *p* = 5e-62). The Hellinger distance between the median and upper limit, excluding outliers, was 0.5141 for the GE group and was 0.2243 for the Siemens group.

## Discussion

In this work, we turn from the characterization of uniformly acquired data to the heterogeneous data of the type available in clinical repositories. In previous work, we used the histogram distance to measure the effects of vendor, site, field strength, and echo time in multi-site data acquired on a small number of subjects with a harmonized protocol. There, we calculated a threshold using simulations of histograms from specific distributions to determine statistical significance of observed differences. This was necessary because there were only five subjects at each site and hence only 15 between-subject distances were available for the within-site distance metric histograms, too few to make statistically-meaningful comparisons between distributions. In the present work, we have more subjects available at each comparison level (e.g., between vendors or with a given number of diffusion-sensitizing gradient directions) and we can statistically measure the similarity of the resulting histogram distance metric histograms.

It can be seen from the representative histograms shown in Figure 1 that neither the FA (Figure 1A) nor the MD (Figure 1B) values are normally distributed. This is not unexpected given the heterogeneous, but ordered nature of brain tissue constituents. This does however point to the need for a method to compare histogram similarity that is sensitive to the complicated shape of the distribution, rather than relying on mean, median, or peak height.

It would be expected that if all diffusion metric volumes were identical except for additive random noise, the shape of the resulting distance metric histograms would be Gaussian. The comparisons were made in a hierarchical fashion (e.g., vendor, vendor at a specific field strength, vendor at a specific field strength with a specific *b*-value, vendor with a specific *b*-value and number of gradient directions) to determine if, at any level on comparison of the distance metric histograms were indeed Gaussian. It was determined that for any of the comparisons we performed the distance metric histograms were always skewed rightward and were more similar to non-normal distributions, such as lognormal, Weibull, or chi-squared (see Figures 2, 3). Part of this shape is likely due to the natural lower bound of zero, but the shape also reflects the natural variability of the underlying acquisition process and data.

The program we adopted here was to suggest a method that potential repository users might use to answer questions that arise when looking to reuse diffusion data (though we emphasize that this method is not specific to diffusion MRI data). For example: what are the fewest number of parameters that one needs to match to get acceptably similar data? Therefore, while it is obvious when looking at Table 1 that there is data with a wide variety of acquisition parameters, one doesn't immediately have a method with which to determine the relative importance of those differences. We suggest that the histogram distance is a particularly good tool for this purpose.

To begin to determine which parameters are important, we first looked at the broadest subset: that of vendor. If one determined that the distance histograms were the same across vendors irrespective of other acquisition parameters, then one could be justified in combining data from all vendors. In the data examined here, the FA and MD maps derived from diffusion data collected on GE scanners had a greater variability than those collected on Siemens scanners (Figure 4). This is somewhat surprising given the distribution of acquisition parameters presented in Table 1. From that data one can see that the GE data was more uniform than the Siemens data: 97% of the GE data were acquired on a 1.5T system, while only 45% of Siemens was and the majority of GE data was acquired using only 2 different numbers of gradient directions and a single *b*-value. The Siemens data was much less uniformly distributed in field strength, *b*-value, and number of gradient directions and one would expect that the greater variation in acquisition protocol would translate to a greater range in histogram distances. On the other hand, the SNR for a well-maintained 3.0T scanner will be greater than that for a 1.5T scanner and this also is expected to contribute to a reduction in histogram distance variability for Siemens data. Therefore, each vendor has variations that the other does not and why we further subdivided the data to determine the relative effect of each subdivision.

In Figure 5, we compared GE vs. Siemens data, but with the extra restriction of b = 1,000 s/mm^2^. As seen in Table 1, all GE data had *b* = 1,000 s/mm^2^ so the data in Figure 5 is the same as in Figure 4. However, the expected behavior occurs for the Siemens data, namely that the width of the distance metric histogram becomes smaller, especially for MD.

As all of our data with a *b*-value of 700 s/mm^2^ came from Siemens machines, we performed our between-*b*-value analysis using the Siemens data (Figure 6). We found that data with a *b*-value of 1,000 s/mm^2^ have a larger distance metric range of observed FA and MD values than data with a *b*-value of 700 s/mm^2^ (Figure 6). Neither variations in echo time, in-plane resolution, nor slice thickness could explain the variability. This difference may be due to the difference in signal-to-noise ratio between the two diffusion weightings, with the *b* = 700 s/mm^2^ data having a higher SNR. The difference in SNR could lead to a more accurate determination for the 30% lower *b*-value. Note also that these two measurements result from different spin pools that survive the dephasing effect of the diffusion gradients. In addition, this is expected given the range of gradient directions in the *b* = 1,000 s/mm^2^ data as seen in Table 1, while the *b* = 700 s/mm^2^ data only occurs with a single (high) number of gradient directions (60). Therefore, it's unsurprising that the *b* = 700 s/mm^2^ data is more similar. These data point to the important role that number of gradient directions plays in data similarity. The FA maps between the two *b*-values were more similar than were the MD. Generally, we find this to be true, and we have found that this is due to the varying amounts of cerebral spinal fluid signal in ventricles and left at the edge of the brain after brain extraction using BET.

Diffusion metric volumes collected on Siemens 3.0T scanners had a larger variability than those with a field strength of 1.5T (Figure 7). This difference also points to the effect of the number of gradient directions on the final similarity as Table 1 shows that there is a larger variation in the number of gradient directions in the 3.0T data than in the 1.5T data. This effect was significant when comparing all 3.0T data to all 1.5T data though the MD KS plot (Figure 7D) shows clearly that the difference is fairly small even though the KS statistic and *p*-value are small. This is a consequence of the large numbers of elements in the histograms and should be taken into account when judging an individual comparison. We looked at echo time, in-plane resolution, and slice thickness as potential confounding variables that could explain this unexpected result but none of these varied enough between the two samples to explain the observed difference.

The final three figures (Figures 8–10) all presented results for cases that were maximally differentiated given the data available in this repository. First, in Figure 8, we showed results for the case in which the field strength is different, but the vendor, *b*-value, and number of gradient directions are the same. Interestingly, when *b*-value and number of gradient directions are controlled, there were only small differences in the cumulative FA histogram (Figure 8C) and somewhat larger differences for MD (Figure 8D). Note that the both the Mann–Whitney U and the KS test statistic for FA, while less than the standard α = 0.05, are close and if one normalized α for the number of comparisons, the difference would not be statistically significant. Even with such a normalization the difference in MD cumulative histograms of the distance metrics would show a difference between the two field strengths with the 3.0T data being more variable for both FA and MD. This is somewhat surprising given that with the *b*-value and number of gradient directions controlled one would expect that the greater SNR available at 3.0T would result in more accurate measurements, which are presumably less variable.

In Figure 9, we showed the results for GE data in which field strength and *b*-value were controlled, but the number of gradient directions were different (either 6 or 25). It is unsurprising that there was a statistically significant difference in the distance metric histograms as 6 is the minimal number of gradient directions for construction of the diffusion tensor and the resulting maps would reflect the reduced map “SNR” that arose from the smaller number of diffusion-weighted images used in their construction.

In Figure 10, we finally showed results for a comparison between vendors in the case that *b*-value (1,000 s/mm^2^), number of gradient directions (30) and field strength (1.5T) was all controlled. Here we saw that there was a clear statistical difference between the two vendors with Siemens data distance metric histogram showing increased similarity of the data than did that for GE.

The data presented here makes the case for the control of several variable when constructing data sets for reuse from repositories in which the data do not arise from a harmonized protocol. In summary we found that: (1) there are significant differences in data similarity with different vendors, (2) the number of gradient directions affects data similarity if there are a wide range of values, but also when there are some data sets with minimal (6) number of gradient directions are included in the data set, (3) when other variables are controlled field strength does not have a large effect on data similarity, (4) *b*-value did have a statistically-significant effect on data similarity.

The fact that the spread of the distribution of histogram distances varies widely based on MRI vendor, *b*-value used, and scanner field strength implies that it is important to characterize the degree of variability in these variables when making comparisons between FA and MD maps or reusing data. One goal of this study was to examine the type of data that can be extracted from clinical repositories suitable for use as “healthy control” data and to then characterize the data in terms of metrics of similarity. That a limited number of comparisons could be done with such a data set shows that data extracted from repositories should be carefully characterized before reuse for other purposes.

This could be used to qualify sites for multi-site trials or used to determine variability after scanner upgrades. We are currently instantiating a web portal that will allow users to compare their uploaded FA or MD volumes to a normative data set by displaying the histogram of distance metrics for the uploaded data set compared to each volume in the normative data set against the similar within-group histogram calculated for the normative data set itself.

## Conclusion

We have presented this histogram-distance-based method to determine the similarity of acquired MRI map volumes. This method can be used most easily on quantitative maps, but can also be used on image-intensity data with suitable normalization. We used this method on data extracted from a clinical repository and showed the process by which this data set was constructed. The results point to the careful curation necessary for the reuse of such data.

## Author contributions

GW contributed to the analysis and interpretation of data. He also contributed to the drafting and revision of the manuscript. KH contributed the conception and design of the work, as well as contributed to the analysis and interpretation of data. He also contributed to the drafting and revision of the manuscript and gave final approval of the published version. KH agrees to be accountable for all aspects of the work in ensuring that questions related to the accuracy or integrity of any part of the work are appropriately investigated and resolved.

### Conflict of interest statement

The authors declare that the research was conducted in the absence of any commercial or financial relationships that could be construed as a potential conflict of interest.

## References

[B1] Bernas T. Asem E. K. Robinson J. P. Rajwa B. (2008). Quadratic form: a robust metric for quantitative comparison of flow cytometric histograms. Cytometry A 73, 715–726. 10.1002/cyto.a.20586 18561196

[B2] Bester M. Jensen J. H. Babb J. S. Tabesh A. Miles L. Herbert J.. (2015). Non-Gaussian diffusion MRI of gray matter is associated with cognitive impairment in multiple sclerosis. Mult. Scler. 21, 935–944. 10.1177/1352458514556295 25392318 PMC4429046

[B4] Cercignani M. Bammer R. Sormani M. P. Fazekas F. Filippi M. (2003). Inter-sequence and inter-imaging unit variability of diffusion tensor MR imaging histogram-derived metrics of the brain in healthy volunteers. AJNR Am. J. Neuroradiol. 24, 638–643. 12695195 PMC8148683

[B5] Cha S.-H. Srihari S. N. (2002). On measuring the distance between histograms. Pattern Recogn. 35, 1355–1370. 10.1016/S0031-3203(01)00118-2

[B7] Cox C. Reeder J. E. Robinson R. D. Suppes S. B. Wheeless L. L. (1988). Comparison of frequency distributions in flow cytometry. Cytometry 9, 291–298. 10.1002/cyto.990090404 3402280

[B10] Duda R. O. Hart P. E. Stork D. G. (2000). Pattern Classification. New York, NY: Wiley-Interscience.

[B11] Farrell J. A. Landman B. A. Jones C. K. Smith S. A. Prince J. L. van Zijl P. C.. (2007). Effects of signal-to-noise ratio on the accuracy and reproducibility of diffusion tensor imaging-derived fractional anisotropy, mean diffusivity, and principal eigenvector measurements at 1.5 T. J. Magn. Reson. Imaging 26, 756–767. 10.1002/jmri.21053 17729339 PMC2862967

[B12] Fox R. J. Sakaie K. Lee J. C. Debbins J. P. Liu Y. Arnold D. L.. (2012). A validation study of multicenter diffusion tensor imaging: reliability of fractional anisotropy and diffusivity values. AJNR Am. J. Neuroradiol. 33, 695–700. 10.3174/ajnr.A2844 22173748 PMC8050446

[B13] Goodyear B. G. Zayed N. M. Cortese F. Trufyn J. Costello F. (2015). Skewness of fractional anisotropy detects decreased white matter integrity resulting from acute optic neuritis. Invest. Ophthalmol. Vis. Sci. 56, 7597–7603. 10.1167/iovs.15-17335 26618652

[B15] Haibin L. Kazunori O. (2006). Diffusion distance for histogram comparison, in Proceedings of the 2006 IEEE Computer Society Conference on Computer Vision and Pattern Recognition - Vol. 1 (Washington, DC: IEEE Computer Society).

[B16] Helmer K. G. Chou M. C. Preciado R. I. Gimi B. Rollins N. K. Song A.. (2016). Multi-site study of diffusion metric variability: characterizing the effects of site, vendor, field strength, and echo time using the histogram distance. Proc. SPIE Int. Soc. Opt. Eng. 9788:97882U. 10.1117/12.2217445 27350723 PMC4919981

[B19] Jenkinson M. Beckmann C. F. Behrens T. E. Woolrich M. W. Smith S. M. (2012). Fsl. Neuroimage 62, 782–790. 10.1016/j.neuroimage.2011.09.015 21979382

[B20] Kang Y. Choi S. H. Kim Y. J. Kim K. G. Sohn C. H. Kim J. H.. (2011). Gliomas: histogram analysis of apparent diffusion coefficient maps with standard- or high-b-value diffusion-weighted MR imaging–correlation with tumor grade. Radiology 261, 882–890. 10.1148/radiol.11110686 21969667

[B22] Lampariello F. (2000). On the use of the Kolmogorov-Smirnov statistical test for immunofluorescence histogram comparison. Cytometry 39, 179–188. 10.1002/(SICI)1097-0320(20000301)39:3<179::AID-CYTO2>3.0.CO;2-I 10685074

[B23] Landman B. A. Farrell J. A. Jones C. K. Smith S. A. Prince J. L. Mori S. (2007). Effects of diffusion weighting schemes on the reproducibility of DTI-derived fractional anisotropy, mean diffusivity, and principal eigenvector measurements at 1.5T. Neuroimage 36, 1123–1138. 10.1016/j.neuroimage.2007.02.056 17532649 PMC12008999

[B24] Long F. Zhang H. Feng D. D. (2003). Fundamentals of Content-based Image Retrieval, in Multimedia Information Retrieval and Management: Technological Fundamentals and Applications, eds Feng D. D. Siu W. C. Zhang H. (Berlin; Heidelberg: Springer-Verlag), 1–26.

[B25] Magnotta V. A. Matsui J. T. Liu D. Johnson H. J. Long J. D. Bolster B. D. Jr.. (2012). Multicenter reliability of diffusion tensor imaging. Brain Connect. 2, 345–355. 10.1089/brain.2012.0112 23075313 PMC3623569

[B28] Nalichowski R. Keogh D. Chueh H. C. Murphy S. N. (2006). Calculating the benefits of a research patient data repository. AMIA Annu. Symp. Proc. 2006:1044. PMC1839563 17238663

[B29] Noether G. (1963). Note on the Kolmogorov statistic in the discrete case. Metrika 7, 115–116. 10.1007/BF02613966

[B30] Nusbaum A. O. Tang C. Y. Wei T. Buchsbaum M. S. Atlas S. W. (2000). Whole-brain diffusion MR histograms differ between MS subtypes. Neurology 54, 1421–1427. 10.1212/WNL.54.7.1421 10751250

[B31] Pagani E. Hirsch J. G. Pouwels P. J. Horsfield M. A. Perego E. Gass A.. (2010). Intercenter differences in diffusion tensor MRI acquisition. J. Magn. Reson. Imaging 31, 1458–1468. 10.1002/jmri.22186 20512899

[B32] Pettitt A. N. Stephens M. A. (1977). The Kolmogorov-Smirnov goodness-of-fit statistic with discrete and grouped data. Technometrics 19, 205–210. 10.1080/00401706.1977.10489529

[B33] Pfefferbaum A. Adalsteinsson E. Sullivan E. V. (2003). Replicability of diffusion tensor imaging measurements of fractional anisotropy and trace in brain. J. Magn. Reson. Imaging 18, 427–433. 10.1002/jmri.10377 14508779

[B34] Poline J. B. Breeze J. L. Ghosh S. Gorgolewski K. Halchenko Y. O. Hanke M.. (2012). Data sharing in neuroimaging research. Front. Neuroinform. 6:9. 10.3389/fninf.2012.00009 22493576 PMC3319918

[B35] Pope W. B. Lai A. Mehta R. Kim H. J. Qiao J. Young J. R.. (2011). Apparent diffusion coefficient histogram analysis stratifies progression-free survival in newly diagnosed bevacizumab-treated glioblastoma. AJNR Am. J. Neuroradiol. 32, 882–889. 10.3174/ajnr.A2385 21330401 PMC7965548

[B36] Pope W. B. Qiao X. J. Kim H. J. Lai A. Nghiemphu P. Xue X.. (2012). Apparent diffusion coefficient histogram analysis stratifies progression-free and overall survival in patients with recurrent GBM treated with bevacizumab: a multi-center study. J. Neurooncol. 108, 491–498. 10.1007/s11060-012-0847-y 22426926 PMC3997502

[B37] Roederer M. Treister A. Moore W. Herzenberg L. A. (2001). Probability binning comparison: a metric for quantitating univariate distribution differences. Cytometry 45, 37–46. 10.1002/1097-0320(20010901)45:1<37::AID-CYTO1142>3.0.CO;2-E 11598945

[B38] Rovaris M. Iannucci G. Cercignani M. Sormani M. P. De Stefano N. Gerevini S.. (2003). Age-related changes in conventional, magnetization transfer, and diffusion-tensor MR imaging findings: study with whole-brain tissue histogram analysis. Radiology 227, 731–738. 10.1148/radiol.2273020721 12702828

[B40] Steffen-Smith E. A. Sarlls J. E. Pierpaoli C. Shih J. H. Bent R. S. Walker L.. (2014). Diffusion tensor histogram analysis of pediatric diffuse intrinsic pontine glioma. Biomed Res. Int. 2014:647356. 10.1155/2014/647356 25006580 PMC4071985

[B41] Takao H. Hayashi N. Kabasawa H. Ohtomo K. (2012). Effect of scanner in longitudinal diffusion tensor imaging studies. Hum. Brain Mapp. 33, 466–477. 10.1002/hbm.21225 21391276 PMC6869949

[B42] Tozer D. J. Davies G. R. Altmann D. R. Miller D. H. Tofts P. S. (2006). Principal component and linear discriminant analysis of T1 histograms of white and grey matter in multiple sclerosis. Magn. Reson. Imaging 24, 793–800. 10.1016/j.mri.2005.08.002 16824974

[B43] Wagner M. W. Narayan A. K. Bosemani T. Huisman T. A. Poretti A. (2016). Histogram analysis of diffusion tensor imaging parameters in pediatric cerebellar tumors. J. Neuroimaging 26, 360–365. 10.1111/jon.12292 26331360

[B45] Yankeelov T. E. Lepage M. Chakravarthy A. Broome E. E. Niermann K. J. Kelley M. C.. (2007). Integration of quantitative DCE-MRI and ADC mapping to monitor treatment response in human breast cancer: initial results. Magn. Reson. Imaging 25, 1–13. 10.1016/j.mri.2006.09.006 17222711 PMC2634832

[B46] Young I. T. (1977). Proof without prejudice: use of the Kolmogorov-Smirnov test for the analysis of histograms from flow systems and other sources. J. Histochem. Cytochem. 25, 935–941. 10.1177/25.7.894009 894009

