# Supplementary material for: Characterization of Diffusion Metric Map Similarity in Data From a Clinical Data Repository Using Histogram Distances
Source: Front Neurosci. 2018 Mar 8;12:133. doi: 10.3389/fnins.2018.00133 (PMC5852401; doi:10.3389/fnins.2018.00133)
Supplement: Supplementary file 1 [file Presentation1.pdf]

Appendix - Histogram Distance Metric Descriptions
Minkowski Metrics
We consider five metrics from the Minkowski family as they are the simplest versions of distance metrics: 1) City Block (Duda et al., 2000) (also known as the L1-norm), Euclidean (Duda et al., 2000) (L2-norm), and Chebyshev (Webb and Copsey, 2011). The City Block metric simply adds up the absolute differences between histogram bins. The Euclidean metric is the familiar distance formula between two points, generalized to the case of histograms. The Chebyshev metric is simply the maximum value of the difference between the histograms for any of the bins. These are some of the simplest metrics, but are not very sensitive to the case in which histograms that may be similarly shaped, but simply offset from each other.

City Block:

$$D_{CB}(A,B) = \sum_{i=0}^{b-1} |H_i\,(A) - H_i(B)| \tag{1a}$$

Euclidean:

$$D_{Euc}(A,B) = \left(\sum_{i=0}^{b-1}(H_i\,(A) - H_i(B))^2\right)^{1/2} \tag{2a}$$

Chebyshev:

$$D_{Che}(A,B) = \max_i |H_i(A) - H_i(B)| \tag{3a}$$

Here *b* is the the total number of bins in the set $x = x_0, x_1, \ldots x_{b-1}$, *n* is the total number of binned elements, and $H_i(X)$ is the frequency of histogram *X* in bin *i*.

The Canberra and Lorentzian metrics are variations of the L1-norm. The Canberra (Webb and Copsey, 2011), which normalizes the absolute differences to the sum of the two bins values and is known to be sensitive to small changes near zero, and the Lorentzian (Deza and Deza, 2012) which is essentially the log of the L1-norm, though unity is added to ensure non-negativity and to avoid the log of zero.

Canberra:

$$D_{Can}(A,B) = \sum_{i=0}^{b-1} \frac{(|H_i(A) - H_i(B)|)}{H_i(A) + H_i(B)} \tag{4a}$$

Lorentzian:

$$D_{Lor}(A,B) = \sum_{i=0}^{b-1} \ln(\,1 + |H_i(A) - H_i(B)|)$$

(5a)

Intersection Family

The non-intersection (Duda et al., 2000) metric is based on the minimum value between the histograms at each bin. Since we are here interested in the differences between histograms we choose the non-intersection, rather than the intersection metric and because we are dealing with normalized histograms, this metric is zero if the histograms are identical. Other, more complicated versions of the Intersection family, such as the Czekanowski (Gordon, 1999) metric can be shown to be equivalent to the simpler non-intersection metric used here in the case of normalized histograms.

Non-Intersection:

$$D_{NI}(A,B) = 1 - \sum_{i=0}^{b-1} \min(H_i(A), H_i(B))$$

(6a)

Fidelity family

This family contains metrics that employ the sum of the modified geometric means of the histograms using the square root rather than the b-th root. We choose the commonly-used Hellinger (Deza and Deza, 2012) metric and the Squared-chord (Deza and Deza, 2012) metric which is the most general version. Other commonly used metrics from this family are the Bhattacharyya (Bhattacharyya, 1943; Choi and Lee, 2003) distance, Matusita (Matusita, 1951; 1955) distance. The Bhattacharyya distance has been shown to be a bound on the Bayesian minimum mis-classification probability and is related in form to the Matusita distance.

Hellinger:

$$D_{Hel}(A,B) = 2[1 - \sum_{i=0}^{b-1} [H_i\,(A) \cdot H_i(B)]^{1/2}]^{\frac{1}{2}}$$

(7a)

Squared-chord:

$$D_{SC}(A,B) = \sum_{i=0}^{b-1} (\sqrt{H_i(A)} - \sqrt{H_i(B)})^2$$

(8a)

Inner product Family

Metrics in the Inner Product family treat the two histograms as vectors and calculate the inner

product normalized by some factor. Here we choose the Cosine (Webb and Copsey, 2011) metric that is the inner product normalized by the square-root of the sum of the squares of each histogram element. This family also contains the familiar Jacquard (Jacquard, 1901) and Dice (Dice, 1945) metrics and which also contain the inner product but have a different normalizing factor.

Cosine:

$$D_{Cos}(A,B) = \frac{\sum_{i=0}^{b-1} H_i\,(A) \cdot H_i(B)}{[\sum_{i=0}^{b-1}(H_i\,(A))^2]^{\frac{1}{2}} \cdot [\sum_{i=0}^{b-1}(H_i\,(B))^2]^{1/2}}$$

(9a)

Squared –L2 Norm

Here we use the squared χ^2 metric (Deza and Deza, 2012) because its normalizing factor is symmetric in the two histograms (as opposed to the Pearson (Deza and Deza, 2012) and Neyman (Deza and Deza, 2012) χ^2 metrics which normalize the squared differences in the numerator by the bin value of one or the other of the histograms). This metric is essentially the normalized Euclidean distance between two vectors.

Squared Chi-Squared

$$D_{SQS}(A,B) = \sum_{i=0}^{b-1} \frac{(H_i(A) - H_i(B))^2}{H_i(A) + H_i(B)}$$

(10a)

Shannon Entropy

These metrics take the form of Shannon's entropy (the quantity $p \times \ln(p)$) with various choices of normalizing factors. When applied to two histograms, it measures the minimum cross entropy of two probability distributions. The metrics in this family are not, in fact, true distances since they are not symmetric with respect to the ordering of the input histograms. We used the Kullback-Leibler (Kullback and Liebler, 1951) distance, and Jeffreys (Jeffreys, 1946) metrics, the latter being the symmetric form of the former.

Kullback-Leibler

$$D_{KL}(A,B) = \sum_{i=0}^{b-1} H_i(A) ln \frac{H_i(A)}{H_i(B)}$$

(11a)

Jeffreys:

$$D_J(A,B) = \sum_{i=0}^{b-1} (H_i(A) - H_i(B)) ln \frac{H_i(A)}{H_i(B)}$$

(12a)

Earth Movers Family

The Earth Mover's distance (EMD) (Rubner et al., 1998) calculates the minimum amount of work that is necessary to transform one distribution to another. We have used the Cha-Srihari (Cha and Srihari, 2002) distance for ordinal data, which is related to the EMD rather than the EMD itself because the Cha-Srihari metric is a special univariate case of the EMD and is of *O(b)* rather than $O(b^3)$ complexity and therefore has a lower computational burden. Note that all the metrics discussed here operate on single-bins except for the Cha-Srihari metric.

Cha-Srihari distance:

$$D_{CS}(A,B) = \sum_{i=0}^{b-1} |\sum_{j=0}^{i} (H_j\,(A) - H_j(B)|$$

(13a)

All of the chosen metrics, except for the K-L distance, are true metrics because they each satisfy the properties of non-negativity (d(xy) ≥ 0), commutativity (d(x,y) = d(y,x)), reflexivity (d(x,x) = 0), and the triangle inequality (d(x,z) ≤ d(x,y) + d(y,z)).

References

Bhattacharyya, A. (1943). On a measure of divergence between two statistical populations defined by their probability distributions. *Bull. Calcutta Math. Soc.* 35**,** 99-109.

Cha, S.-H., and Srihari, S.N. (2002). On measuring the distance between histograms. *Pattern Recognition* 35(6)**,** 1355-1370.

Choi, E., and Lee, C. (2003). Feature extraction based on the Bhattacharyya distance. *Pattern Recognition* 36(8)**,** 1703-1709.

Deza, M., and Deza, E. (2012). *Encyclopedia of Distances.* Springer.

Dice, L. (1945). Measures of the Amount of Ecologic Association Between Species. *Ecology* 26**,** 297–302.

Duda, R.O., Hart, P.E., and Stork, D.G. (2000). *Pattern Classification.* New York: Wiley-Interscience.

Gordon, A. (1999). *Classification.* Chapman and Hall/CRC.

Jacquard, P. (1901). Étude comparative de la distribution florale dans une portion des Alpes et des Jura. *Bulletin de la Société Vaudoise des Sciences Naturelles* 37**,** 547–579.

Jeffreys, H. (1946). An invariant form for the prior probability in estimation problems. *Proc R Soc Lond A Math Phys Sci* 186(1007)**,** 453-461.

Kullback, S., and Liebler, R.A. (1951). On information and sufficiency. *Ann. Math. Statist.* 22**,** 79-86.

Matusita, K. (1951). On the theory of statistical decision functions. *Ann. Inst. Stat. Math* 3**,** 17-35.

Matusita, K. (1955). Decision rules, based on the distance, for problems of fit, two samples and

estimation. *Ann. Math. Statist.* 26**,** 631-640.
Rubner, Y., Tomasi, C., and Guibas, L.J. (Year). "A metric for distributions with applications to image databases", in: *Computer Vision, 1998. Sixth International Conference on*), 59-66.
Webb, A., and Copsey, K. (2011). *Statistical Pattern Recognition.* Wiley.
